# A personalized and automated real-time meal detection algorithm based on continuous glucose monitoring and heart rate data for individuals with post-bariatric hypoglycemia

**DOI:** 10.3389/fdgth.2026.1758474

**Published:** 2026-07-01

**Authors:** Luca Cossu, Giacomo Cappon, Felix Wortmann, David Herzig, Lia Bally, Andrea Facchinetti

**Affiliations:** 1 Department of Information Engineering, University of Padova, Padova, Italy; 2 Universität St. Gallen, Zürich, Switzerland; 3 Department of Diabetes Endocrinology Nutritional Medicine and Metabolism Inselspittal, Bern University Hospital University of Bern, Bern, Switzerland

**Keywords:** continuous glucose monitoring (CGM), heart rate, meal detection, meal event, real-time, smartwatch

## Abstract

**Introduction:**

Continuous glucose monitoring (CGM) sensors are increasingly used to identify and manage post-bariatric hypoglycemia (PBH) and to support decision support systems (DSSs) for proactive glucose management. Despite meal timing being key information for these systems, automated, wearable-based meal detection remains an unmet clinical need.

**Methods:**

We present a real-time meal detection algorithm for individuals with PBH that combines CGM and heart rate (HR) signals. The algorithm is a heuristic decision-tree model based on four individualized features extracted from CGM (rate of change, glucose relative excursion, glucose peak value) and HR (peak value). It was developed and tested using a dataset of 40 PBH patients monitored for up to 50 days with a Dexcom G6 CGM and a Garmin Venu Sq smartwatch, and its performance was evaluated in both controlled and free-living conditions, benchmarked against state-of-the-art CGM-only meal detection methods for the PBH population.

**Results:**

The algorithm achieved 100% recall in the controlled setting and, in free-living conditions, an average precision of 85% and recall of 78%. It also reduced false positives compared with CGM-only algorithms (one every 2.3 days vs. one every 1.3 days).

**Discussion:**

Eliminating the need for manual meal announcement, the proposed algorithm overcomes a key barrier to fully automated glucose management, reducing patient burden while maintaining reliable detection performance even in unstructured, free-living conditions. These results support the integration of the algorithm into DSSs for PBH and other populations, where timely and accurate meal detection is critical.

## 1 Introduction

Meal detection plays a pivotal role in various domains, including medicine, psychology, and clinical research (1). For example, the timing of meals significantly impacts glucose metabolism and is essential for understanding glycemic patterns, adjusting therapies, and managing conditions where glucose regulation is disrupted. In this work, we focus on individuals affected by post-bariatric hypoglycemia (PBH), a metabolic complication of bariatric surgery (2, 3). The condition is characterized by meal-induced glucose surges, followed by rapid glucose declines and episodes of hypoglycemia (4). PBH arises as a complication in some patients after bariatric surgery, driven by exaggerated insulin responses to carbohydrate ingestion. In the absence of approved and effective pharmacotherapies, dietary modification (e.g., reduction of rapid-acting carbohydrates) remains the cornerstone of PBH management (5). In this context, continuous glucose monitoring (CGM) sensors have emerged as valuable tools for monitoring glucose fluctuations and predicting hypoglycaemia (5). These sensors provide real-time data on glycemic trends, enabling both patients and clinicians to make informed decisions regarding behavioral adaptations, including meal modifications. In people with insulin-treated diabetes requiring information of meal onset is crucial for optimal timing of insulin dosing, particularly when striving for fully automated artificial pancreas systems (6, 7).

However, the accurate identification of meal timing remains a critical unmet need in this population, as manual logging of meals is burdensome and prone to inaccuracies, such as missed entries or misattributions (6).

Automated meal detection techniques have been mainly developed using CGM data from people with type 1 diabetes (T1D) (7). For instance, Ramkissoon et al. (8) and Turksoy et al. (9) recently presented two algorithms aimed at automatically identifying meal times from CGM signal to inform artificial pancreas systems using Kalman filters, while more complex techniques such as neural networks have been explored by Mosquera-Lopez et al. (10) for that population.

On the other hand, despite the potential for improving the management of PBH in clinical practice, previous work on automated meal detection algorithms in this population remains limited. To date, the most notable contribution is the work of Laguna-Sanz et al. (11), who developed a CGM-based meal detection algorithm aimed at predicting and mitigating PBH using automated glucagon delivery (12).

Furthermore, although CGM sensors hold promise for meal detection algorithms, these methods encounter notable obstacles (1). Firstly, the physiology of glucose absorption introduces a delay between meal ingestion and detectable glycemic excursions in CGM data (13). Secondly, CGM sensors measure glucose in the interstitial fluid, which lags behind blood glucose levels by several minutes (14). Finally, the standard sampling rate of CGM devices, typically every five minutes, may not capture rapid glucose fluctuations with sufficient resolution. Additional confounding factors, such as glucose changes due to exercise, stress, or low-carbohydrate meals, further complicate the task of reliable meal detection when using the CGM as a stand-alone signal (15).

In order to overcome these limitations, additional meal-related signals acquired from sources other than CGM can be investigated (6). For instance, wearable devices, such as smartwatches, offer a promising avenue for augmenting CGM data. Among the signal these devices collect, heart rate (HR) has shown potential for meal detection, as meal intake induces physiological responses (16), including increased HR due to blood redistribution to the gastrointestinal tract (17, 18). Notably, HR changes occur immediately after meal onset, with peak levels observed approximately 15 min post-ingestion (19). Combining CGM data with HR measurements, it is possible to develop algorithms that leverage the complementary strengths of these modalities to improve the accuracy and timeliness of meal detection.

In this study, we aim to develop a personalized algorithm tailored to the PBH population for automated real-time meal detection. Our approach integrates CGM data with HR monitoring from smartwatches to overcome the limitations of CGM-based detection alone.

## 2 Methods

### 2.1 Datasets

In this work, we used two datasets generated by forty post-bariatric surgery patients (Roux-en-Y gastric bypass) who were fitted with the Dexcom G6 CGM device and the Garmin VenuSQ smartwatch. Patients (age 48.8 ± 13.3 years, BMI 26.7 ± 5.2 kg/m^2^) underwent surgery more than two years prior the enrolment and had non-diabetic hemoglobin A1c (HbA1c) of 5.4 ± 0.4%. In this population, accelerated glucose absorption leads to more discernable meal-induced glucose and potentially also HR peaks, supporting the early algorithmic development and analysis work.

The first dataset contained supervised meal events from 40 in-clinic visit sessions with a standardized solid mixed meal test (bread with butter and jam, and fruit yogurt, comprising 584 kcal, 85 g of carbohydrates, of which 40 g of sugar, 21 g of fat, 12 g of protein) (20) and documented time of meal onset. This dataset was used to validate the assumption of HR level increase during mealtime.

The second dataset was generated in the same population but during 7 weeks of unrestricted living during an observational clinical study. During this period, meal events were self-recorded by the participants using the study mobile application and retrospectively verified and further labelled by the research team by a standardized meal event assignment procedure, as previously reported (21). Specifically, a meal event was labelled when two independent researchers independently marked the same event based on a selection criterion of a glucose increment of at least 18 mg/dL over 60 min followed by a subsequent decrease. Only events marked concordantly by both researchers and fulfilling the predefined glucose excursion criterion were retained as ground truth.

A total of 7,031 meal events has been labelled in this dataset, with an average of 3.5 ± 1.4 meals per day. In both datasets, CGM signals were collected every five minutes while the heart rate signal was sampled at a 30-second interval.

Both datasets used in this work were collected with written informed consent of study participants within the framework of clinical studies (NCT05216926, NCT05212207), approved by the local Ethics Committee (Project ID 2021–02086 Kantonale Ethikkommision Bern, Switzerland).

### 2.2 Verification of meal-related alterations of the HR signal

To verify the presence of the meal-related rise in HR we used the in-clinic dataset. This was performed by extracting the peak in the HR signal with a minimum value of 65 beats/min. This threshold was chosen on the basis of a resting heart rate of 60bpm in the baseline resting condition in which patients were during the visit. The peak search was applied within 20 min before and after the meal onset mark. Once HR peaks within the time of interest were identified, we quantified the distance between the peak and the meal onset and evaluated the distribution of these time distances.

Following the alignment of all HR signals with meal onset marks logged during the controlled visits, we analyzed the signal distribution and shape around mealtime, exploring the average pattern of the heart rate signal in the mealtime window.

### 2.3 Meal detection algorithm overview

The algorithm is a rule-based decision tree, which leverages on domain knowledge with hand-crafted features and was first developed on separate 10 days not included in the free-living dataset.

**Figure 1:**
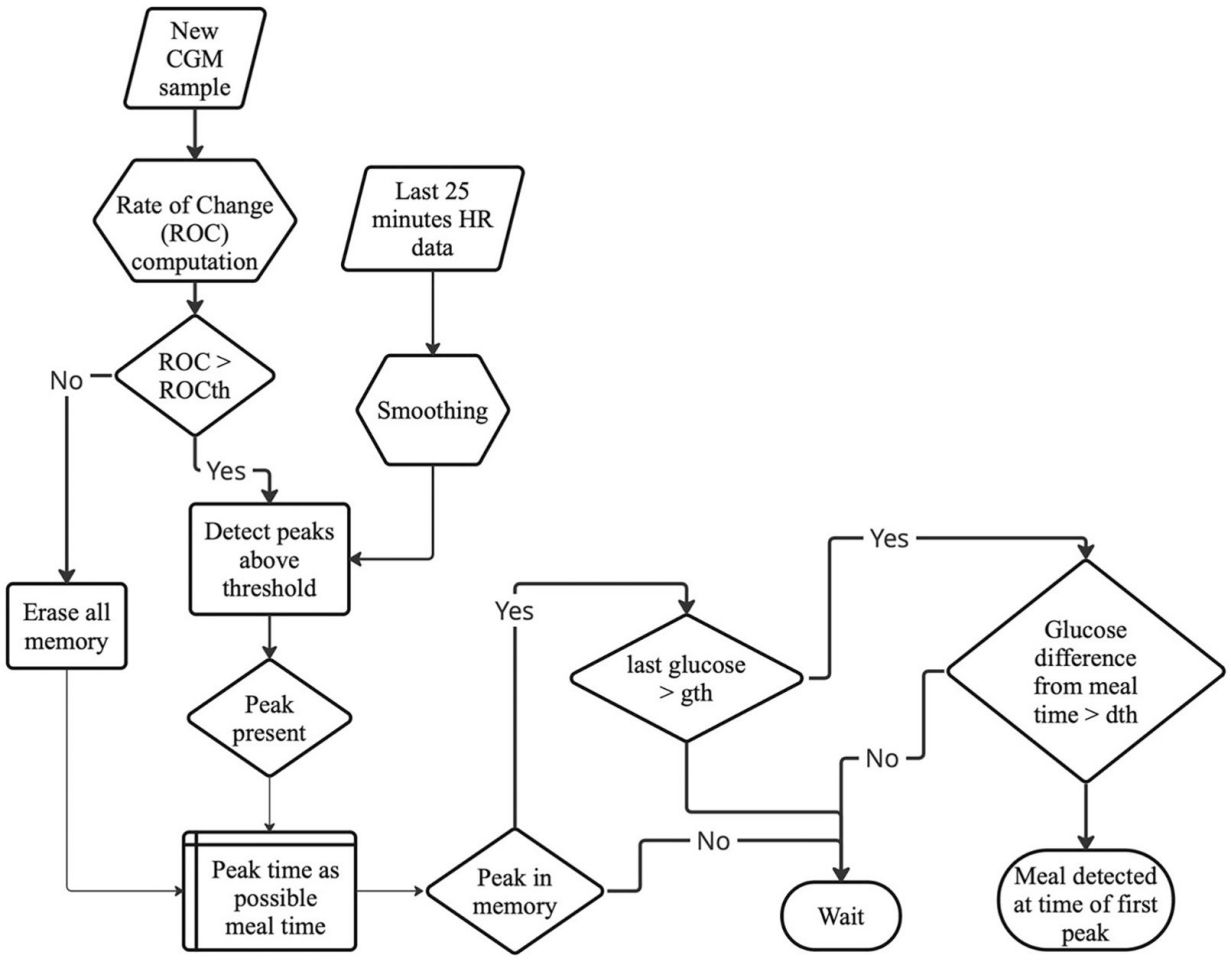
Schematic representation of data flow in the algorithm. Each time a new CGM sample is collected, its rate of change (ROC) is calculated. If the ROC is higher than the ROC threshold value (ROCth), the last 25 min of HR data are smoothed and then analyzed, searching for peaks above the HR threshold (HRth). If a peak is found, it is saved as possible mealtime, and the current glucose value is stored. The next iteration if the peak is confirmed, the current glucose value and the difference from the one at mealtime are compared with two thresholds, gth and gdth respectively. If both values are above the respective thresholds, the first detected peak is marked as meal.

The algorithm workflow, illustrated in Figure 1, is triggered at each new CGM sample collection. In the first step, the algorithm computes the rate of change (ROC) of the CGM signal over the preceding 15 min as $ROC=\frac{CGM_{t}-CGM_{t-15}}{15}$.

If this rate surpasses the ROC threshold (ROCth = 0.3 mg/dL/min), the second step is initiated: the algorithm searches for peaks in the HR signal over the preceding 25 min. To enhance accuracy, the HR signal is first preprocessed with a moving average over 5 samples to reduce noise.In the third step, if an HR peak above a threshold (HRth = 70 beats/min) is identified, its timestamp is stored as a potential meal candidate and the current glucose value is recorded. In the fourth step, at the next CGM iteration, the algorithm repeats the ROC and HR peak assessment: if a peak is confirmed again, the candidate is promoted to a strong meal candidate. In the fifth and final step, the algorithm verifies the candidate by checking whether the current glucose value exceeds a threshold (gth = 130 mg/dL) and whether the glucose increment from the stored baseline exceeds a second threshold (gdth = 30 mg/dL). If both conditions are met, the meal event is confirmed. All these population-level thresholds’ values were determined resorting to an iterative optimization procedure performed in the free-living dataset. Specifically, candidate thresholds and temporal windows were systematically varied within physiologically plausible ranges. For example, a resting heart rate of 60 bpm and an average glucose increase after a meal of at least 30 mg/dL were assumed. Performance metrics (precision, recall, F1-score, see section 2.5) were evaluated at each step. The selected final thresholds correspond to those maximizing overall performance at the population level before subject-specific personalization.

If at any point the ROC becomes negative or no HR peak is found, the algorithm resets to its initial state. The two glucose thresholds (gth, gdth) serve to filter out detections caused by minor glucose fluctuations or non-meal-related perturbations. The algorithm operates in real-time, but can also be applied retrospectively by processing each time step using only the data available up to that point.In case of missing data of either CGM or HR signal the algorithms remains silent.

### 2.4 Personalization procedure

Recognizing that the initially set values may not universally accommodate individuals with varying resting heart rates, post-meal peak glucose levels, or unique characteristics, we employ a gradient search optimization procedure to personalize the algorithm parameters for each subject. This personalized approach aims to enhance the algorithm's precision and applicability across diverse patient profiles.

he four thresholds introduced in Section 2.3 (ROCth, HRth, gth, and gdth) constitute the parameter vector TH targeted by the optimization. For each subject, the optimization is performed on data from the first 14 days of the free-living recording, using the F1-score as the cost function. The gradient-based search operates within physiologically motivated bounds for each parameter. The F1-score is defined as: $F1=\frac{2\times \mathrm{Precision}\times \mathrm{Recall}}{\mathrm{Precision}+\mathrm{Recall}}$. The optimization problem is thus defined as $TH_{opt}=\underset{TH}{argmin}\;(F1(TH))$.

The resulting personalized parameter set is then applied without modification to the remaining recording days, which serve as the held-out evaluation set. This temporal separation between the optimization and evaluation phases ensures that reported performance reflects generalization to unseen data, rather than fitting to the calibration period.

### 2.5 Performance evaluation

To assess algorithm performance, we evaluated the temporal proximity of detected meal event to the actual meal onset time (our reference).

The metrics used for evaluation are precision and recall.

To calculate these metrics, a true positive detection was considered valid if the detected meal occurred within a window of 20 min before or after the actual meal event. This threshold has been set considering multiple factors, such as the average delay between a meal event and a visible CGM increase [considering both glucose absorption (∼10 min) and intrinsic CGM delay (∼10 min) (22, 23)], and the average duration of a meal event, which has been estimated during the in-clinic visits.False positive events were defined as meal detections outside the defined 20-minute window. False negative events were considered meal events that were not detected by the algorithm. To compensate for large increases in glucose levels not related to labelled meals, the false positives where the presence of a glucose peak above 180 mg/dL in the subsequent hour is identified, are excluded from performance computations, and marked as removed false positives. It is important to note that, in the PBH population, an extensive glucose increase is likely related to a meal event. Thus, the aim being to correctly evaluate algorithm performance, we removed these false positive, relative to missed labelled meals. Although this adjustment aims to avoid penalizing the algorithm for likely missed annotations, it may lead to a slight overestimation of precision, which should be considered when interpreting results.

Performance was evaluated in both datasets, i.e., in the controlled setting (observed ground-truth of meal ingestion) and in the dataset generated in free-living conditions, calculating the per patient metrics and then reporting the average. In the free-living dataset, the metrics have been computed on the data not used for personalizing the parameters, while in the in-clinic dataset no optimization procedure was performed.

### 2.6 Comparison with two state-of-the art meal detection algorithms using CGM alone in PBH

To further evaluate the algorithm's performance, we compared the proposed combined CGM-HR meal detection algorithm with two algorithms using only CGM data. The first, called ROCdet in this work, considers a meal when the ROC value surpasses a threshold in two subsequent samples, similarly to Dassau et al. (24) for T1D patients. The second comparator has been proposed by Laguna Sanz et al. (11) for the post-bariatric population and modifies the ROCdet algorithm by marking a meal when three subsequent smoothed ROC values are above 1 mg/dL/min. The latter is referenced as LS in this work. A Wilcoxon rank sum test has been performed to statistically compare the performance of the algorithm in comparison with the comparators. A *p*-value < 0.05 was considered statistically significant.

### 2.7 Code availability

The code implementing the proposed algorithm and personalization procedure, and evaluation pipeline is available at github.com (repository link to be updated upon publication)

## 3 Results

### 3.1 In-clinic dataset results

This section presents the results obtained on the in-clinic dataset, where meal intake was directly supervised. The controlled setting of these experiments allows a rigorous assessment of the algorithm's core detection performance under known ground-truth conditions.

#### 3.1.1 Verification of the HR peak presence around mealtime

**Figure 2:**
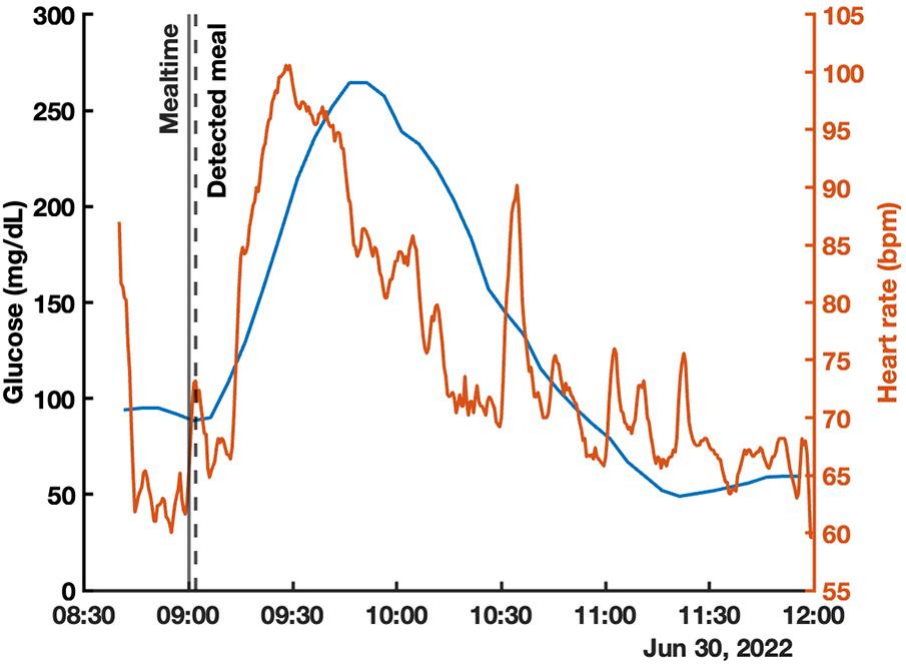
Representative example of a heart rate (HR) trace around a supervised meal event (in-clinic dataset). The top panel shows glucose (blue) and HR (red) signals. The vertical solid line marks the meal onset; the vertical dashed line indicates the corresponding HR peak detected by the algorithm, illustrating the typical postprandial HR elevation occurring within minutes of ingestion.

Using the supervised data from in-clinic visits with supervised meal intake, we observed HR peaks usually occurring 1 min in median after the true meal onset, with 25th and 75th percentiles of 0 and 5 min respectively. In 6 of the 40 sessions, the peak was identified before the mealtime and in 2 sessions the peak lagged more than 10 min behind meal onset. A representative glucose and HR trace is shown in Figure 2, where the vertical dashed line is the HR peak in correspondence to the meal event (vertical solid line).

#### 3.1.2 Algorithm performance in detecting meals

**Figure 3:**
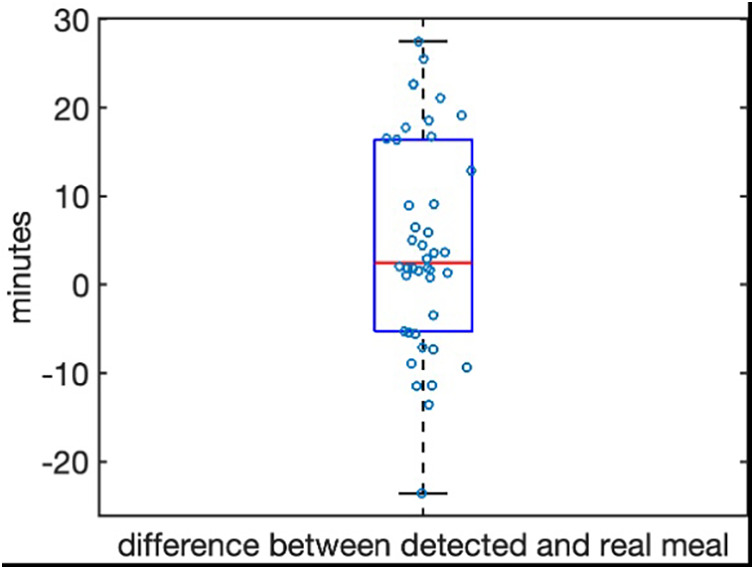
Distribution of the time difference (in minutes) between algorithm detection and true meal onset in the in-clinic controlled setting. Positive values indicate detections occurring after meal onset; negative values indicate early detections. Box plot shows median and interquartile range; individual subject data points are overlaid with horizontal jitter.

The median difference between the detection and the mealtime (shown in Figure 3) was 1 min, with the algorithm detection occurring later than the actual one. With the 25th and 75th percentiles of −5 and 15 min respectively, the deviation was less or equal to the average duration of the meal event itself, which was 13 min on average. The distribution of the difference in the 10 min range can be implied to different physiological responses to the meal which was not accounted for with a fixed threshold. In the situation of meal detections lagging more than 20 min behind the actual onset, HR signal traces consistently had been collected only few minutes before mealtime, and no relevant peak was identified around mealtime.

The overall recall of the algorithm was above 92% considering the temporal proximity of 20 min for a true positive, while false negatives (i.e., missed detection) is associated to missing data in either the HR or the CGM signal shortly before the meal. Precision in the in-clinic dataset is 100%. On average, the algorithm triggered the detection of a meal with a mean delay of 25 min from the actual onset of the meal.

### 3.2 Free-living conditions dataset results

This section reports the results obtained on the free-living dataset, where participants recorded meal events autonomously over seven weeks. The evaluation includes the personalization of algorithm parameters to individual physiology, the overall detection performance, and a comparison with state-of-the-art CGM-only approaches.

#### 3.2.1 Setting algorithm parameters to account for individual physiology and meal patterns

**Figure 4:**
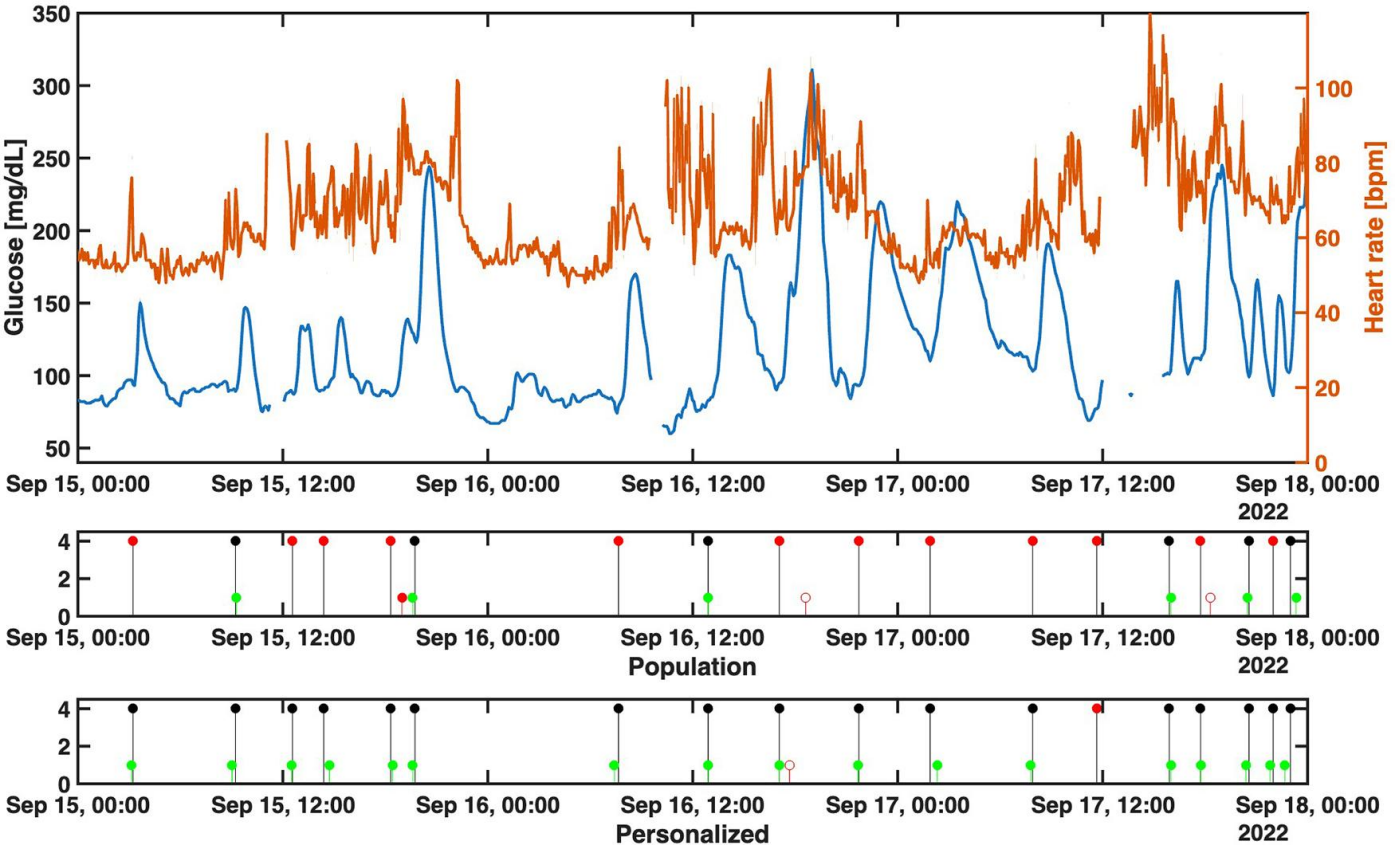
Effect of the personalization on a sample subject in the dataset (focus on three days). On the top chart, the glucose and heart rate (HR) signals are depicted in blue and red, respectively. In the two panels below, the detected events by the proposed algorithm are shown with the following color-coding: logged meal events (black stem and dots); false negative detections (black stem and red dot); true positives (green stem and dot); false positives (red stem and dot). The middle panel shows the detections by the algorithm with the population parameters (6 true positives and 12 false negatives), in the bottom panel the personalized algorithm detections are shown (17 true positives and 1 false negative).

As designed, the algorithm comprises four parameters that can be adjusted to account for individual characteristics related to physiology and behavior. The tuning of these parameters is essential when considering a dataset spanning 7 weeks, as certain patients exhibit comparably lower resting HR than others during this time window. Consequently, the threshold regarding the HR peak at mealtimes need to be lower than the standard value used in the in-clinic phase. Figure 4 provides an illustrative example of the impact of the individualization of the parameters. Utilizing the population thresholds, as depicted in the middle chart, the algorithm recognizes a mere 6 meal events while failing to identify 12 events within the specified three-day timeframe. In contrast, the individualized algorithm correctly discerns 17 meal events, with only one oversight. Modifying the threshold to accommodate individual characteristics facilitates the detection of most events (represented as a green stem in the lower two charts). Conversely, the generic algorithm parameters fail to capture numerous events due to the subject's low average HR signal.

#### 3.2.2 Personalized algorithm performance in free-living conditions

**Figure 5:**
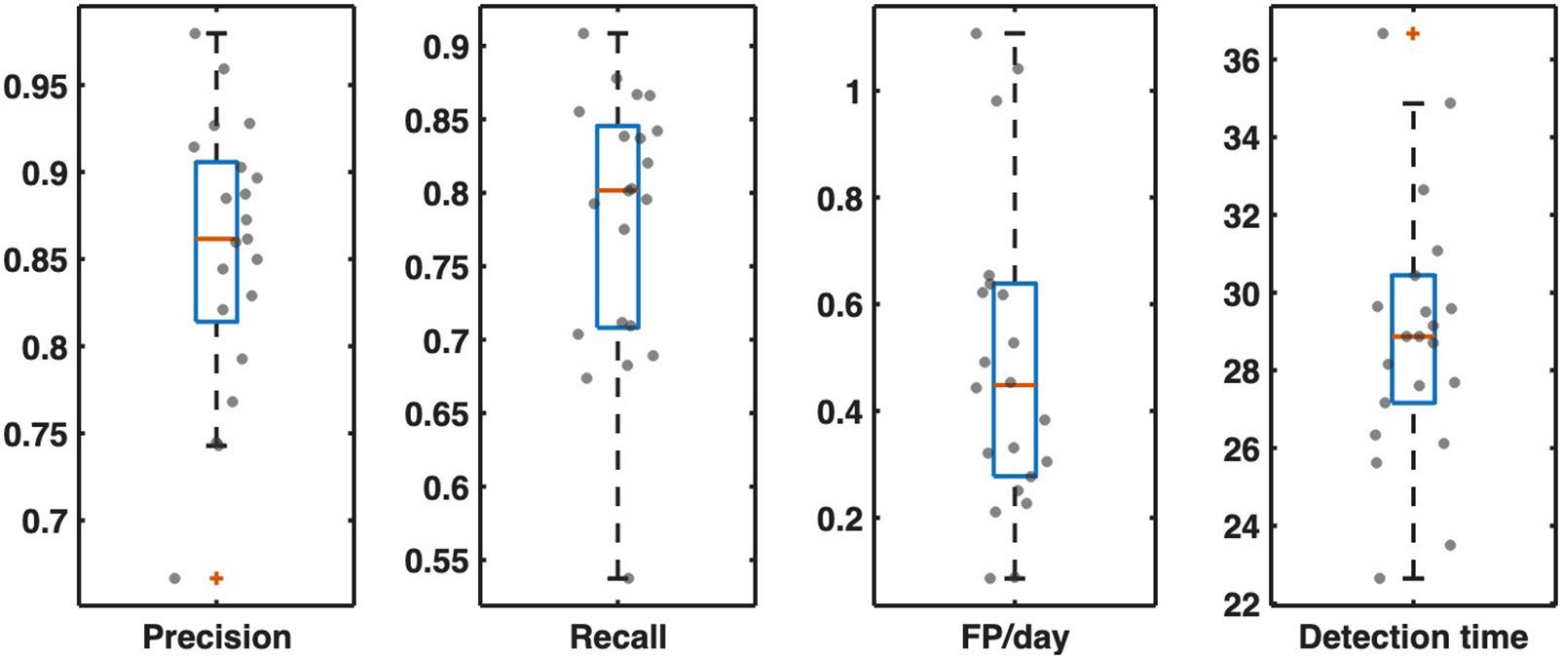
Performance of the proposed personalized algorithm on the full free-living dataset. The detection time refers to the difference in minutes between the meal event and the time when the algorithm confirms the detection. Individual subject data points are overlaid on each box plot with horizontal jitter.

The personalized algorithm showed a median precision of 85% and a recall of 78% on the free-living dataset, with interquartile range (IQR) of 12% and 13% respectively. On average, the algorithm triggers a false positive every two days of data, and the median detection time (difference between meal onset to detection) was 28 min (IQR of 3 min). These results are shown in Figure 5 for the full dataset.

**Figure 6:**
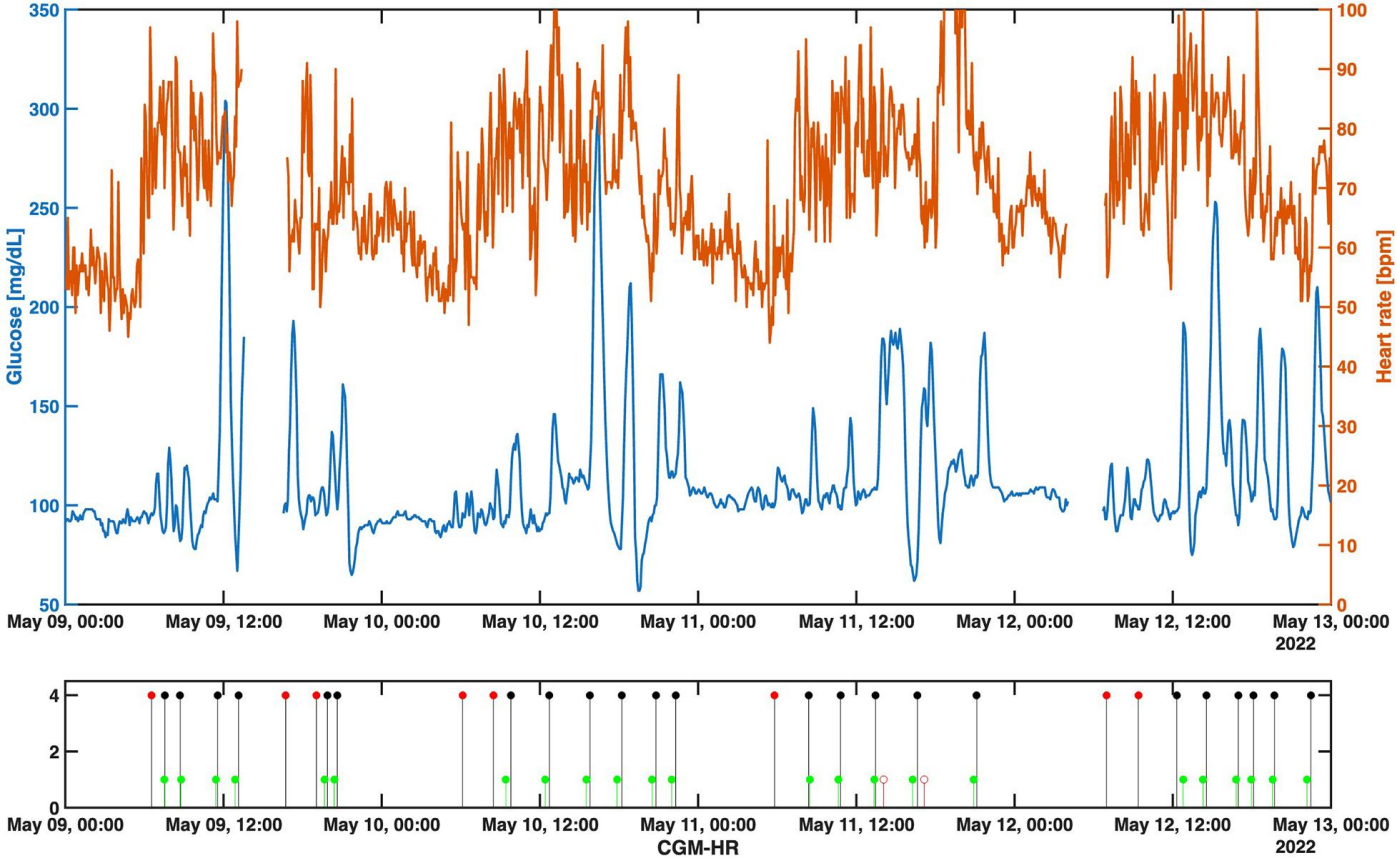
Meal detection performance example in the free-living conditions (zoom on 4 days). On the top chart, the glucose and heart rate (HR) signals are depicted in blue and red, respectively. In the bottom panel, the detected events by the proposed algorithm are shown with the following color-coding: actual meal events (black stem and dots); false negative detections (black stem and red dot); true positives (green stem and dot); false positives (red stem and dot).

An individual's data example is provided in Figure 6, which illustrates the glucose and HR signals over 4 days, accompanied by a visualization of actual and algorithm-detected meal events. For this subject, the proposed algorithm yielded a recall and a precision of 90% and 87% respectively, resulting in one false positive every 2 days, with a mean detection time of 25 min.

#### 3.2.3 Comparison with state-of-the-art CGM-only algorithms

**Figure 7:**
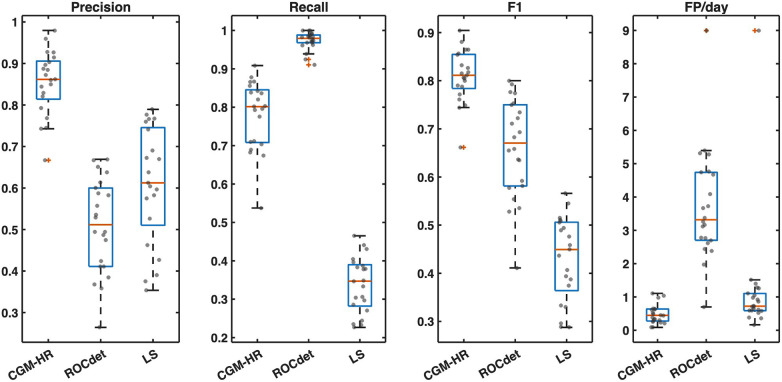
Comparative plots of performance metrics between the three algorithms considered. CGM-HR is the proposed algorithm, which overall performs better than the comparators (ROCdet and LS). CGM-HR outperforms both the comparators in terms of precision and number of false positives per day, while having a median recall of 78% (IQR of 13%), only lower than the recall of ROCdet. The F1-score of the CGM-HR algorithm is significantly higher that the comparators. ROCdet is the gold standard algorithm based on CGM rate of change; LS is the algorithm proposed by Laguna Sanz tailored to a similar population.

The results of the comparative analysis between the algorithms are shown in Figure 7. Overall, CGM-HR outperforms both comparators. In fact, CGM-HR has a higher F1-score (*p*-value < 0.05) than the two comparators. In detail, CGM-HR exhibits higher precision (*p*-value < 0.05) compared to CGM-only algorithms, but lower recall than ROCdet algorithm. Lower recall is, however, offset by the significantly lower false positives per day achieved with the CGM-HR algorithm compared with the other algorithms (*p*-value <0.05 for both comparisons).

**Figure 8:**
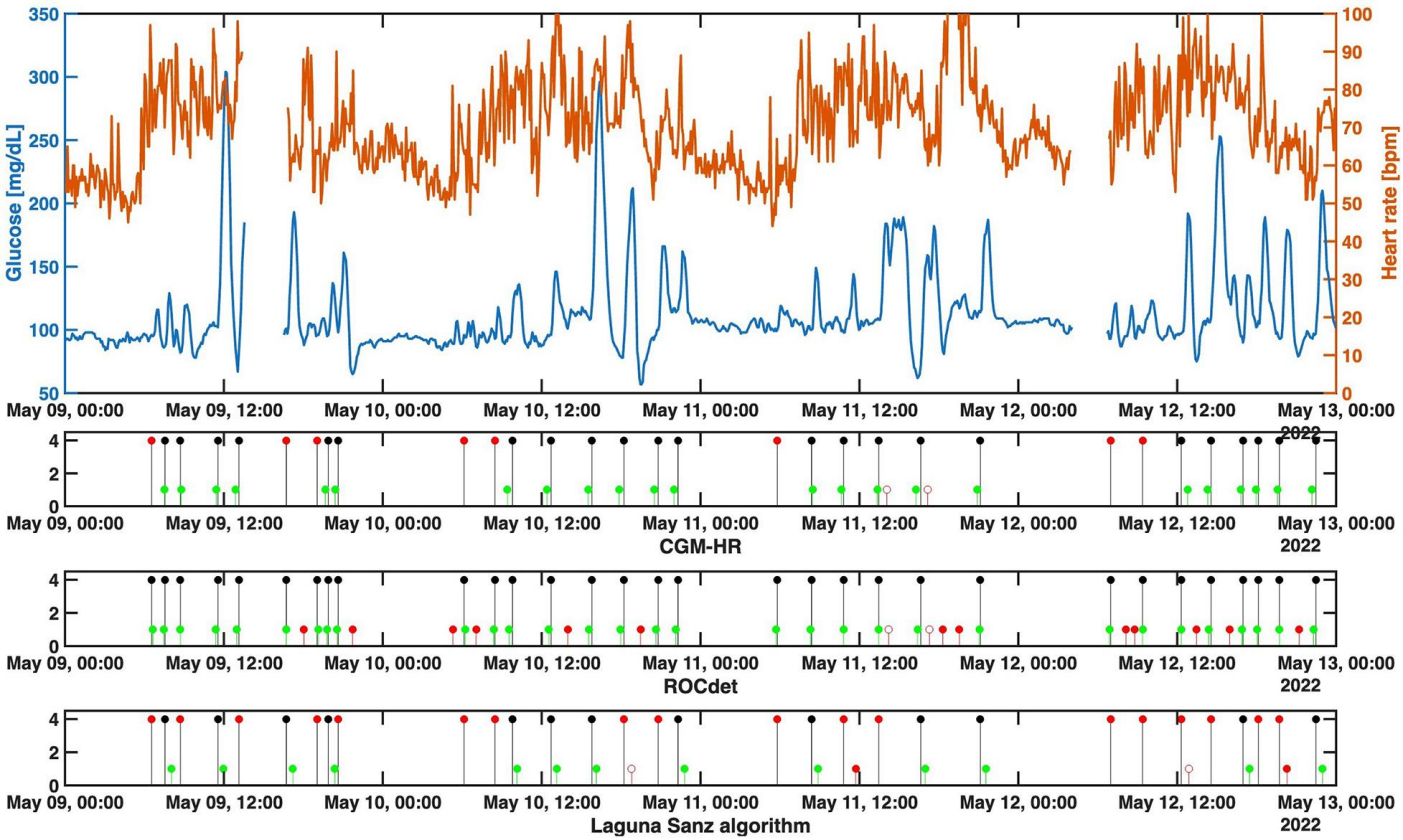
Example of comparison of the meal detection performance of the three considered algorithms. Color-coding: actual meal events (black stem and dots); false negative detections (black stem and red dot); true positives (green stem and dot); false positives (red stem and dot). The proposed CGM-HR algorithm on the second panel is able to detect the majority of the events, and when compared to the other two algorithms it can be seen how the ROC algorithm in the third panel triggers many false positives and the Laguna-Sanz algorithm in the bottom panel has many false negatives.

In Figure 8, we illustrate the comparison of the three algorithms on the basis of an individual data example. Compared to the CGM-HR algorithm, the ROCdet algorithm demonstrates a better recall performance (all events correctly identified), but a greater number of false positives per day (more than 10 in the four-day window shown). Compared to the LS algorithm, the CGM-HR shows a lower number of missed meal events, with only 8 missed events compared to 18 of the LS algorithm. Table 1 summarizes the metrics for the compared methodologies.

**Table 1:** Summary of metrics evaluated in the free-living dataset. Metrics are reported as median and IQR. Detection time is the difference between the trigger of the detection and the detected event.

Method	Precision (%)	Recall (%)	F1-score	Detection time (min)	False positives per day
ROCdet	53 [21]	98 [3]	67 [19]	20 [2]	3.3 [2]
CGM-HR	78 [13]	85 [12]	82 [8]	28 [3]	0.5 [0.4]
LS	60 [27]	35 [11]	45 [13]	26 [2]	0.7 [0.5]

## 4 Discussion

In this work, we propose a real-time algorithm for the meal-time detection in individuals affected by PBH that exploits HR and CGM data acquired via wearable technologies. The algorithm was developed and validated in a dataset generated by a representative sample of PBH patients wearing the Dexcom G6 CGM and the Garmin VenuSQ smartwatch during controlled and free-living conditions. Following an iterative development process, we first provided evidence of a meal-related HR peak in the controlled dataset and then used the combined HR and CGM signals to provide a first version of an automated meal detection algorithm (CGM-HR). We then used the dataset acquired in free-living conditions to further optimize and personalize the algorithm. Median recall and precision were 92% and 100% in the controlled environment and 78% and 95% in free-living. Meal-related HR signals in combination with CGM reduced the false discovery rate compared to state-of-the-art methods based solely on CGM data for the PBH population.

The proposed approach is the first exploratory use of the HR signal from a smartwatch as an add-on to CGM data to automate meal event detection, thereby obviating the need for manual input for the PBH population. Previous HR-related works were largely focused on estimating physical activity (25–27). The use of HR signals to improve meal detection has been preliminarily explored in the T1D population through the integration of physical-activity physiological models to extend glucose–insulin models for identifying meal-related glucose excursions (28). In that context, HR was primarily incorporated as an adjunctive signal within a physical exercise modeling framework, as outlined by Breton et al., rather than as a primary driver of meal detection. Additional approaches include the detection of “about-to-eat” or emotional eating episodes using multivariate machine-learning models not specifically related to metabolic conditions (29, 30), as well as retrospective meal identification strategies (31). Additional approaches for meal detection have relied smartwatch-generated movement reflecting hand gestures (32). However, these existing methods each exhibit limitations: CGM-only algorithms in this population often exhibit high false discovery rates due to the non-specific nature of glucose excursions; HR-only or movement-based techniques lack metabolic specificity and are sensitive to confounders such as physical activity or emotional arousal. In contrast, the proposed algorithm deliberately integrates HR and CGM to exploit complementary physiological signatures. Furthermore, unlike machine learning-based approaches, the proposed algorithm is a fully interpretable rule-based system: each decision threshold has a direct physiological rationale, and the detection logic is entirely traceable without the need for *post-hoc* explainability methods. The comparative analysis with two CGM-only state-of-the-art methods in this population indicates that this multi-signal strategy is particularly effective in reducing false detections.

The proposed approach can be adopted prospectively, in real-time for meal-dependent therapeutic decision making or retrospectively, for meal event assignment in existing datasets. While automated meal event detection has the potential to significantly support the management of PBH, various other health conditions may benefit from the approach. Apart from insulin-treated diabetes, where automated meal detection is primarily pursued to develop fully automated artificial pancreas systems, meal detection algorithms may aid eating behavior therapy, e.g., adherence monitoring and detection of deviation from intervention goals. Consequently, personalized intervention could be deployed more proactively. A possible avenue in the field of behavioral psychology, are Just-In-Time Adaptive Interventions (JITAIs) which aim to exploit the full potential of remote monitoring combined with delivering intervention in receptive situations (33). Indeed, In terms of detection delay, our algorithm's performance is comparable to more complex neural-network-based approaches developed for T1D (10), while offering improved reliability for JITAI systems compared to methods relying on manual user input, which are error-prone and increase burden.

The median detection delay of approximately 25–30 min is primarily driven by physiological glucose absorption dynamics, intrinsic CGM latency, and confirmation logic within the decision tree. While this delay, comparable with other state of the art methodologies, may limit ultra-early therapeutic interventions, the algorithm remains suitable for real-time supportive applications and retrospective annotation. Notably, HR peaks were observed close to meal onset in controlled conditions, suggesting potential avenues for latency reduction in future refinements.

Although the approach is novel, the research remains at an early stage and is not without limitations. While the algorithm was initially developed using a controlled dataset with direct observation of ingestive activity, refinement required an additional 50-day free-living dataset. This step was essential for advancing and personalizing the method, yet self-recorded meals and retrospective ground-truth assignment inherently introduce uncertainty compared with real-time observation. While the standardized labeling procedure reduces annotation variability, residual uncertainty inherent to free-living self-reported data cannot be fully excluded. Such variability may influence both threshold learning and performance estimates and should be addressed in future validation studies.

**Figure 9:**
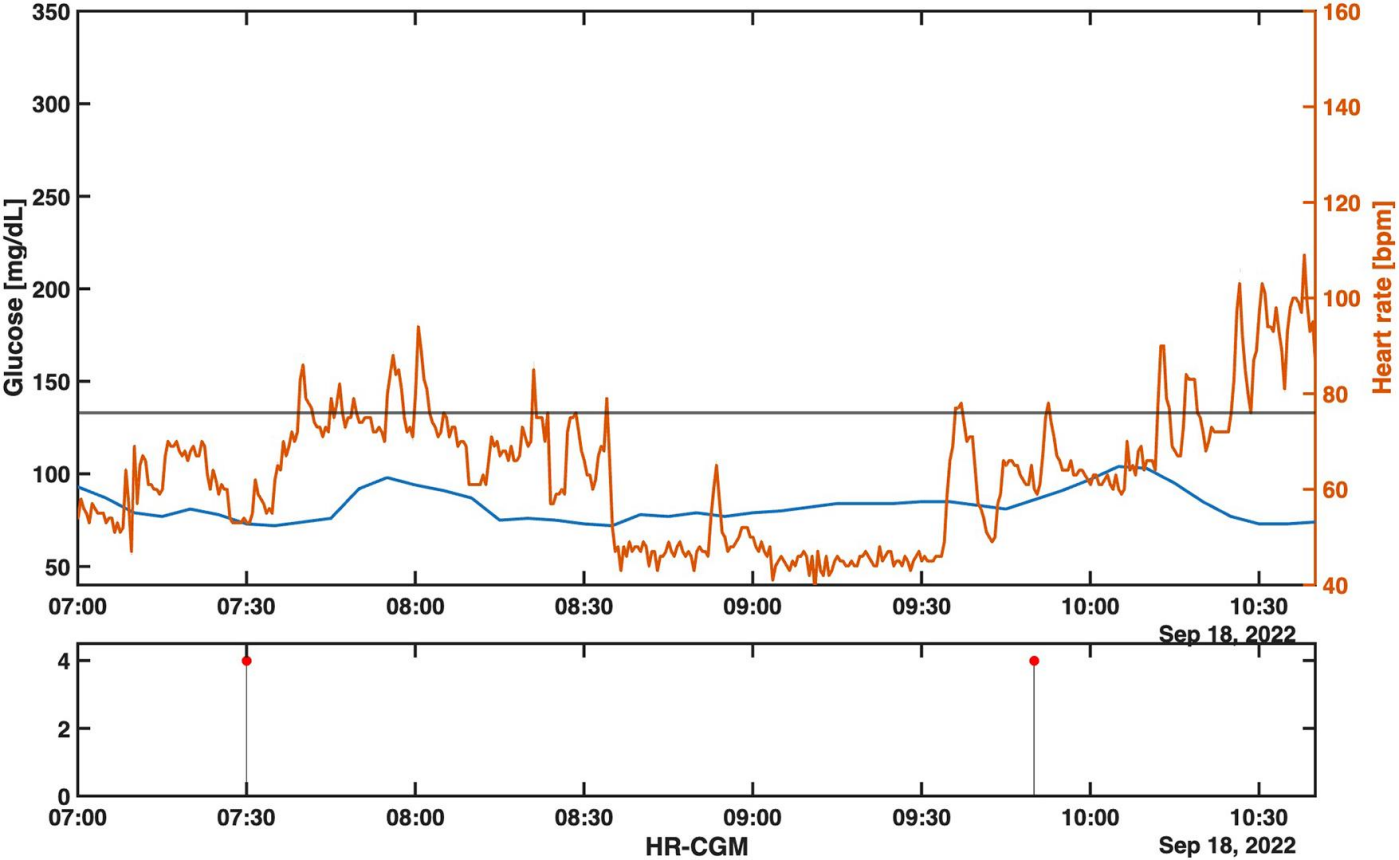
Portion of data in which the CGM-HR algorithm misses the detection of meals. The horizontal dashed line represents the optimized HRth parameter for the patient. It can be seen how in all instances the HR peak does not surpass the threshold, causing the missed detection.

A further limitation relates to the HR signal itself, which is sensitive to factors unrelated to meal intake, including medication effects, physical activity, and emotional stress. The current implementation does not explicitly account for these confounders. Moreover, a single HR related threshold might not completely consider the full daily HR dynamic, leading to wrong or missed detections due to high difference in HR dynamics in resting or active state. Specifically, as illustrated in Figure 9, a resting state event might be missed because the associated HR peak doesn't exceed the HRth parameter. This parameter is optimized across the entire set of daily meals and many of these events likely occur during active periods. Future work will explore using over-basal HR thresholds to compensate for these missed events. Similarly, CGM traces may be perturbed by non-meal-related influences such as signal artefacts or pressure-induced attenuation. The proposed multimodal approach mitigates these risks through the joint evaluation of glucose rate-of-change, amplitude thresholds, and HR dynamics, thereby reducing false detections compared to CGM-only methods. However, neither HR nor CGM perturbations are explicitly modeled, and robustness under defined HR-modulating or CGM-modulating conditions remains to be systematically verified. Future validation in populations with different behavioral patterns, comorbidity profiles, or medication regimens will be necessary to assess generalizability. The need to wear an additional device may also introduce cost and burden, although the widespread uptake of smartwatches in the general population mitigates this concern and supports acceptability in daily use.

Regarding future real-world deployment, the requirement for a 14-day personalization period represents another technical constraint. This step is currently necessary to adapt the algorithm to individual physiology, yet its duration and stability across diverse user groups warrant further evaluation. Introducing continuous adaptation of population parameters during this period may reduce the burden while preserving functionality, but the effectiveness of this strategy has yet to be demonstrated. Indeed, an extensive evaluation of the algorithm in a dedicated clinical trial is required to demonstrate the feasibility of on-device real-time detection of meals in a free-living environment. Long-term stability warrants dedicated evaluation to determine whether the initial personalization remains effective over extended periods, and to assess the potential need for adaptive or continuously updated personalization strategies that account for physiological drift and behavioral changes over time.

Moving forward, we will expand the proposed methodology to other populations, beginning with T1D individuals who already use CGM as part of their routine care. Indeed, the algorithm was developed in people who underwent bariatric surgery, a population characterized by altered postprandial physiology: surgical rearrangement of the gastrointestinal tract produces distinct glucose and HR profiles, including faster and higher glucose excursions and more pronounced postprandial HR increments (34). Although these dynamics are more accentuated than in non-operated individuals, similar qualitative glucose trends are expected in broader populations. Combined with the personalization step implemented in the algorithm, this suggests that the approach may accommodate substantial physiological variability. Nonetheless, feasibility and acceptable performance outside the bariatric setting must be demonstrated before generalization can be reliably assumed.

Overall, this study provides evidence that exploiting the HR signal from a commercial off-the-shelf smartwatch in addition to CGM, can improve the performance of automated meal detection algorithms, particularly with respect to the reduced the false discovery rate. While current evidence is limited to individuals after bariatric surgery, successful extension to other populations could substantially advance the field of digital diagnostics and therapeutics.

## Data Availability

The raw data supporting the conclusions of this article will be made available by the authors, without undue reservation.
